# Effectiveness of the flash glucose monitoring system in preventing severe hypoglycemic episodes and in improving glucose metrics and quality of life in subjects with type 1 diabetes at high risk of acute diabetes complications

**DOI:** 10.1007/s00592-024-02298-x

**Published:** 2024-06-04

**Authors:** Alessandra Dei Cas, Raffaella Aldigeri, Giulia Bellei, Davide Raffaeli, Paolo Di Bartolo, Alessandra Sforza, Giulio Marchesini, Anna Vittoria Ciardullo, Valeria Manicardi, Maurizio Bianco, Marcello Monesi, Anna Vacirca, Maria Cristina Cimicchi, Paola Anna Sordillo, Mattia Altini, Federica Fantuzzi, Riccardo C Bonadonna, for the Flash-glucose monitoring Emilia Romagna Regional network, Maria Grazia Magotti, Silvia Haddoub, Elena Turola, Clelia Di Seclì, Diletta Ugolotti, Francesca Marchignoli, Maria Letizia Petroni, Gilberto Laffi, Rossella D’Urso, Elena Malchiodi, Elisa Manicardi, Lisa Bonilauri, Rita Manini, Costanza Farabegoli, Patrizia Scolozzi

**Affiliations:** 1 Department of Medicine and Surgery, Division of Nutritional and Metabolic Sciences, Azienda Ospedaliero-Universitaria di Parma, Via Gramsci 14, 43126 Parma, Italy; 2 Department of Medicine and Surgery, Università di Parma, Parma, Italy; 3 Diabetes Unit, Azienda Unità Sanitaria Locale (AUSL) Romagna, Ravenna, Italy; 4 Endocrinology Unit, Azienda AUSL Bologna, Bologna, Italy; 5 IRCCS Azienda Ospedaliero-Universitaria di Bologna, Bologna, Italy; 6 Unit of Internal Medicine-Diabetology, Azienda USL Modena, Modena, Italy; 7 Diabetes Clinic, Azienda USL-IRCCS di Reggio Emilia, Reggio Emilia, Italy; 8 Azienda Unità Sanitaria Locale (AUSL) Piacenza, Piacenza, Italy; 9 Primary Care Department, Diabetes Unit, Ferrara ‚‘‘Sant’Anna” Hospital, Ferrara, Italy; 10 Azienda Unità Sanitaria Locale (AUSL) Imola, Imola, Italy; 11 Azienda Unità Sanitaria Locale (AUSL) Parma, Parma, Italy; 12 Hospital Care Sector Manager, Direzione Generale Cura della Persona, Salute e Welfare, Bologna, Italy; 13 Division of Endocrinology and Metabolic Diseases, Azienda Ospedaliero-Universitaria di Parma, Parma, Italy

**Keywords:** Type 1 diabetes, Intermittent-scanned continuous blood glucose monitoring, Severe hypoglycemia

## Abstract

**Aims:**

To assess the effectiveness of the intermittent-scanned continuous glucose monitoring (isCGM) system in preventing severe hypoglycemic episodes and in improving glucose parameters and quality of life.

**Methods:**

Four hundred T1D individuals were enrolled in a prospective real-word study with an intermittently scanned continuous glucose monitoring device during the 12-months follow-up. The primary endpoint was the incidence of severe hypoglycemic events.

**Results:**

82% of subjects were naïve to the use of the device (group A) and 18% were already wearing the system (group B). The cumulative incidence of severe hypoglycemia (SH) at 12 months was 12.06 per 100 person-year (95% CI: 8.35–16.85) in group A and 10.14 (95% CI: 4.08–20.90) in group B without inter-group differences. In group A there was a significant decrease in SH at 12 months compared to 3 months period (*p* = 0.005). Time in glucose range significantly increased in both groups accompanied with a significant decrease in glucose variability. HbA1c showed a progressive significant time-dependent decrease in group A. The use of the device significantly improved the perceived quality of life.

**Conclusion:**

This study confirmed the effectiveness of the isCGM in reducing hypoglycemic risk without glucose deterioration, with potential benefits on adverse outcomes in T1D individuals.

*Trial registration*: ClinicalTrials.gov registration no. NCT04060732.

**Supplementary Information:**

The online version contains supplementary material available at 10.1007/s00592-024-02298-x.

## Introduction

Type 1 diabetes (T1D) accounts for almost 10% of overall diabetes with a growing 3–5% annual incidence [1]. Intensive glucose management delays the onset and slows the progression of microvascular [2] and macrovascular long-term complications [3] with (fear of) hypoglycemia remaining a major limiting factor. Hypoglycaemia not only negatively affects quality-of-life [4] but (severe) hypoglycemic episodes (SHE) are associated with 3.4-fold increased risk of death [5, 6]. Intermittent-scanned continuous glucose monitoring (isCGM) system -Free Style Libre 1 (FSL1)- was introduced as a large-scale alternative to conventional capillary blood glucose monitoring (CBGM). Real-time glucose levels and, importantly, trends can be obtained -on demand- every minute for 14 days and data are automatically stored every 15 min but no glucose alarm is provided, differently from the more recent isCGM FSL2-and FSL3 device which were not available at the time of our study.

In randomised controlled trials (RCT) in T1D, FSL-1 resulted more effective in reducing time spent in hypoglycaemia compared to CBGM without deteriorating hemoglobin A1C (HbA1C) levels [7–9]*,* increased the time spent in target glucose ranges and ameliorated glucose control compared to CBGM [8] and showed a significant reduction in the incidence of mild hypoglycemia and an increased treatment satisfaction, without HbA1c modification [10].

This efficacy in highly controlled settings might no longer be detectable in the usual clinical practice in which a large amount of non-controlled factors may negatively interfere. Most of the real-world evidence (RWE) studies show the effectiveness of the FSL-1 system in ameliorating glucose metrics [11, 12] and acute diabetes events [13].

This study was undertaken to assess the effectiveness of the isCGM system in preventing SHE and in improving glucose parameters and quality of life (QoL) in adult subjects with T1D with a recent history at high risk of acute diabetes complications

## Methods

### Study design

This is a prospective multicenter longitudinal real-world study conducted in 10 diabetes primary care outpatient clinics in the Emilia-Romagna Region, Italy, between May 2017 and December 2018.

### Study population

Inclusion criteria were age ≥ 18 years, T1D according to American Diabetes Criteria (ADA) [14], fasting peptide-C levels ≤ 0.2 nmol/L, disease duration ≥ 12 months, insulin therapy with multiple daily injections (MDI), experience of at least one SHE [15] and/or recurrent documented glucose capillary values < 45 mg/dl (2.5 mmol/L) or hospital admission for diabetic ketoacidosis (DKA) in the last 12 months. Exclusion criteria were pregnancy, type 2 or secondary diabetes, continuous subcutaneous insulin infusion (CSII) therapy or previous use of real-time CGM.

Eligible subjects were divided in new isCGM (FreeStyle Libre® 1- Abbott Diabetes Care) users (naïve patients) (group A) and individuals already using the device out of pocket (group B). All subjects were provided with standard education to the use of the device.

Demographic, anthropometric, and main biochemical/metabolic parameters at baseline and every 3 for 12 months data were collected. Data on microvascular and macrovascular complications were recorded. The validated diabetes-specific QoL in T1D questionnaire [16, 17] was administered at baseline and at 12 months.

### Study endpoints

The primary endpoint was the incidence of SHE defined according to ADA criteria as an event requiring assistance of another person to actively administer carbohydrates, glucagon, or take other corrective actions [15] during the 12 months. Secondary endpoints included plasma HbA1c levels assessed at baseline and every 3 months and changes in some of the standardized continuous glucose-based metrics [18] namely *i*. time per day within target glucose range (TIR) (70–180 mg/dL; 3.9–10.0 mmol/L), time below target glucose range (TBR) level 1 (< 70 mg/dL; < 3.9 mmol/L), and level 2 (< 54 mg/dL; < 3.0 mmol/L) and time above target glucose range (TAR) level 1 (> 180 mg/dL; > 10.0 mmol/L) and level 2 (> 250 mg/dL; > 13.9 mmol/L); *iii* glicemic variability assessed by percentage coefficient of variation (%CV) calculated by standard deviation/mean*100; Glucose; *iv* Management Indicator (GMI). Treatment satisfaction was assessed by changes in Diabetes DQOL score from baseline to study end.

Other secondary endpoints included daily scan frequency, adherence to CGM use (the percentage of captured sensor data between study visits), number of days isCGM worn, number of sensor detachments and of allergic patch reactions throughout the study period. Of note continuous glucose-based metrics and data were relative to the 14 days for V1 and to 3-month period for the other follow-up visits.

## Statistical analysis

Continuous variables are reported as means and standard deviations (SD) or median and interquartile range (IQR), and categorical variables as frequencies and percentages.

The chi-square test was used to evaluate associations between categorical variables while Student t-test or Mann–Whitney test in continuous variables. Spearman correlation coefficient was used to evaluate correlation among variables. Cumulative incidence and incidence rates with Poisson 95% Confidence Intervals were calculated for SHE.

General Linear Model (GLM) for repeated measures was performed to evaluate group and time effect for continuous outcomes and regression model to identify independent predictors of HbA1c change. Differences in glucose metrics during follow-up were compared by Kruskall-Wallis and Wilcoxon signed rank test.

The analysis was conducted according to Intention-to-treat approach.

The analyses were performed using SPSS v.28 (IBM SPSS Statistics), all tests were two-tailed, and a *p*-value ≤ 0.05 was considered statistically significant.

## Results

### Study population

**Fig. 1:**
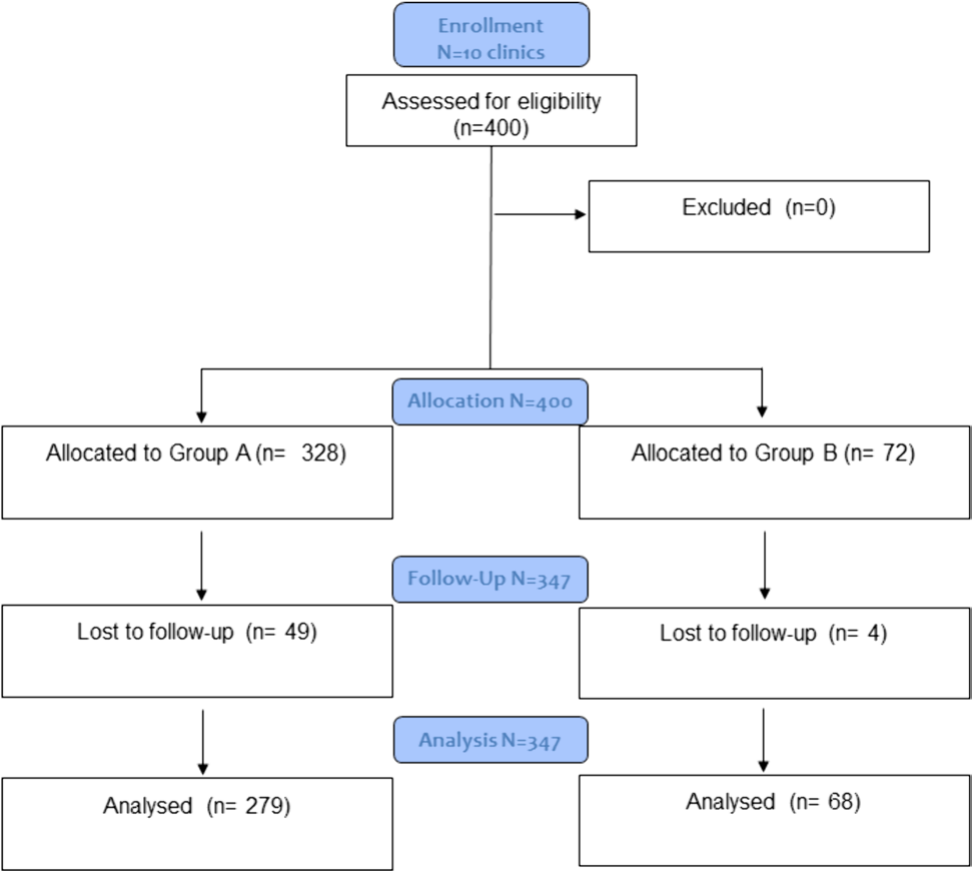
Study CONSORT flow chart. Group A: naive user for FGM device; Group B: former user for FGM device

A total of 400 consecutive T1D individuals were recruited; 328 (82%) naïve (group A) and to 72 (18%) (group B) were already using the system out of pocket (Fig. 1). Main baseline demographic and clinical characteristics of the study individuals by treatment group are shown in Table 1. The two groups did not differ for the main features except for HbA1c which, was higher in the naïve isCGM group and c-HDL levels which were lower in group A compared to group B (Table 1).

**Table 1:** Baseline demographics and clinical characteristics of diabetic patients (n = 400). Results are expressed as mean ± SD, median (IQR) or n (%)

	Missing	Overall (n = 400)	Group A (n = 328)	Group B (n = 72)	*p*-value
Age (years)	0	44 ± 14	44 ± 13	45 ± 15	0.76
Gender, male	0	197 (49.3%)	166 (50.6%)	31 (43.0%)	0.33
Disease duration (years)	0	19.0 (11.0–31.0)	19.5 (12.0–31.0)	19.0 (8.0–27.8)	0.23
BMI (kg/m2)	2	23.4 (21.5–26.2)	23.5 (21.6–26.2)	23.0 (21.1–26.2)	0.69
SBP (mmHg)	0	120 (110–130)	120 (110–130)	120 (110–130)	0.39
DBP (mmHg)	0	70 (70–80)	70 (70–80)	73 (70–80)	0.25
HbA1c (%)	0	7.6(6.8–8.6)	7.6(6.9–8.6)	7.2(6.5–8.3)	0.02
HbA1c (mmol/mol)	0	60 (51–70)	60 (52–70)	55 (47–67)	0.02
Creatinine (mg/dl)	4	0.8 (0.7–0.9)	0.80 (0.70–0.91)	0.76 (0.67–0.90)	0.06
Total cholesterol (mg/dl)	17	180 (161–203)	179 (160–202)	181 (165–204.8)	0.37
HDL cholesterol (mg/dl)	27	59 (50–69)	57 (50–69)	65.5 (57.2–72)	0.001
Triglycerides (mg/dl)	20	72 (55–100)	73.5 (56.2–101.8)	66.5 (54.2–88)	0.08
C-peptide (nmol/L)	13	0.10 (0.01–0.10)	0.10 (0.01–0.10)	0.10 (0.01–0.10)	0.43
Normoalbuminuria n,% Microalbuminuria n,% Macroalbuminuria n,%	0	335 (83.8%) 46 (11.5%) 14 (3.5%)	276 (84.1%) 35 (10.7) 12 (3.7%)	59 (81.9%) 11 (15.3%) 2 (2.8%)	0.47
Prior MI n,%	0	16 (4%)	13 (4.0%)	3 (4.2%)	0.80
Prior stroke n,%	0	2 (0.5%)	2 (0.6%)	0 (0%)	0.64
Foot amputation n,% Foot ulcer n,% Previous foot ulcer n,%	0	1 (0.3%) 1 (0.3%) 11 (2.8%)	1 (0.3%) 1 (0.3%) 9 (2.7%)	0 (0,0%) 0 (0,0%) 2 (2.8%)	0.91
Background RD n,% Preproliferative RD n,% Proliferative RD n,%	0	36 (9%) 24 (6%) 57 (14.2%)	26 (7.9%) 20 (6.1%) 46 (14.0%)	10 (13.9%) 4 (5.5%) 11 (15.2%)	0.50
Maculopathy n,%	0	10 (2.5%)	8 (2.4%)	2 (2.8%)	0.29
CHO counting	3	213(53.2%)	161(49.1%)	48(66.7%)	0.008
Antihypertensive drugs n,%	0	84 (21%)	64 (19.5%)	20 (27.8%)	0.25
Lipid-lowering drugs n,%	0	91 (22.8%)	72 (21.9%)	19 (26.4%)	0.22

BMI, Body Mass Index; SBP, Systolic Blood Pressure; DBP, Diastolic Blood Pressure; RD, diabetic retinopathy, MI, myocardial infarction

At baseline, 25 subjects (6.2%) had a history of DKA 18 (5.5%) in group A and 7 (9.7%) in group B.

### Severe hypoglicemic episodes

**Fig. 2:**
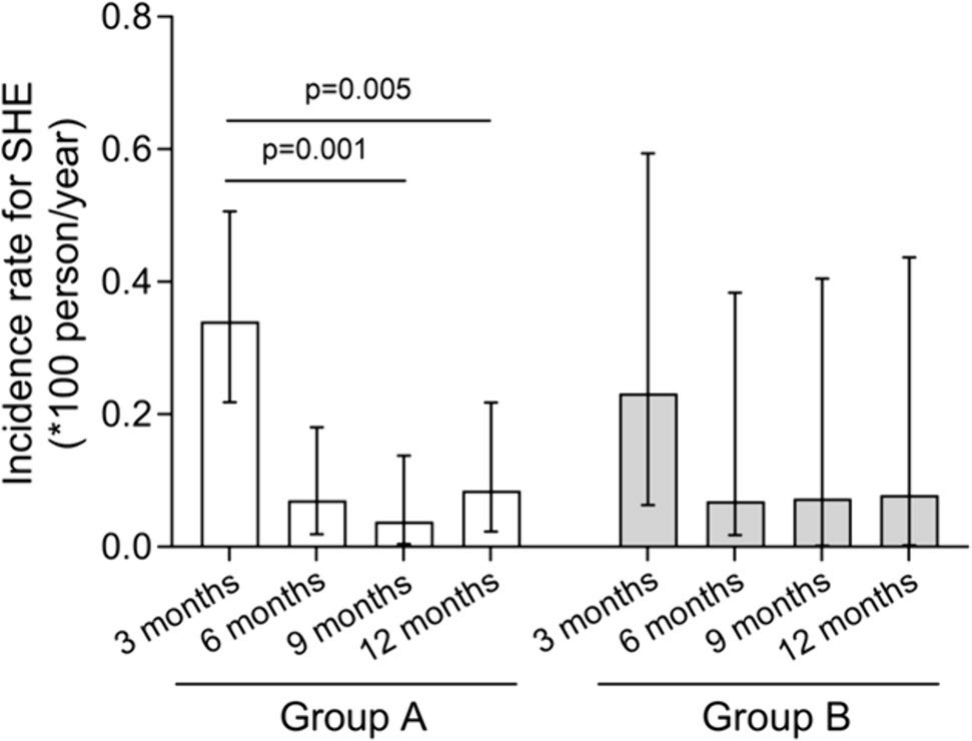
Incidence rates *100 person year for severe hypoglycaemia events (SHE) in the 2 groups (Group A-naive user for FGM device, white bars; Group B-former user for FGM device, gray bars) over time (3 to 12 months). Data plot represent incidence rates and bars show 95% confidence intervals

The cumulative incidence of SHE at 12 months in the whole cohort was 11.7 (95% CI: 8.38–15.85) *100 person-year (41 subjects experienced ≥ 1 SHE); 12.06 (95% CI: 8.35–16.85) in group A and 10.14 (95% CI: 4.08–20.90) in group B without inter-group differences (*p* = 0.67). In group A there was a significant decrease in SHE incidence rate at 12 months compared to 3 months (incidence rate difference 25.5*100 person-year; 95% CI 7.51–43.5 *p* = 0.005), while in group B the rate difference was 15.35*100 person-year, 95% CI − 14.2–44.9 (*p* = 0.31) (Fig. 2). No DKA events occurred during follow-up. In the whole population, at a multivariate analysis, after adjusting for gender, age, study group, and other variables associated at the univariate analysis, higher baseline plasma HbA1C (p = 0.035, OR 1.47), higher CV% (*p* = 0.013, OR 1.06), presence of CHO counting (p = 0.001, OR 0.20) and the outpatients center (*p* < 0.001), were independent predictors of cumulative incidence of SHE (data not shown).

### Metabolic parameters

**Fig. 3:**
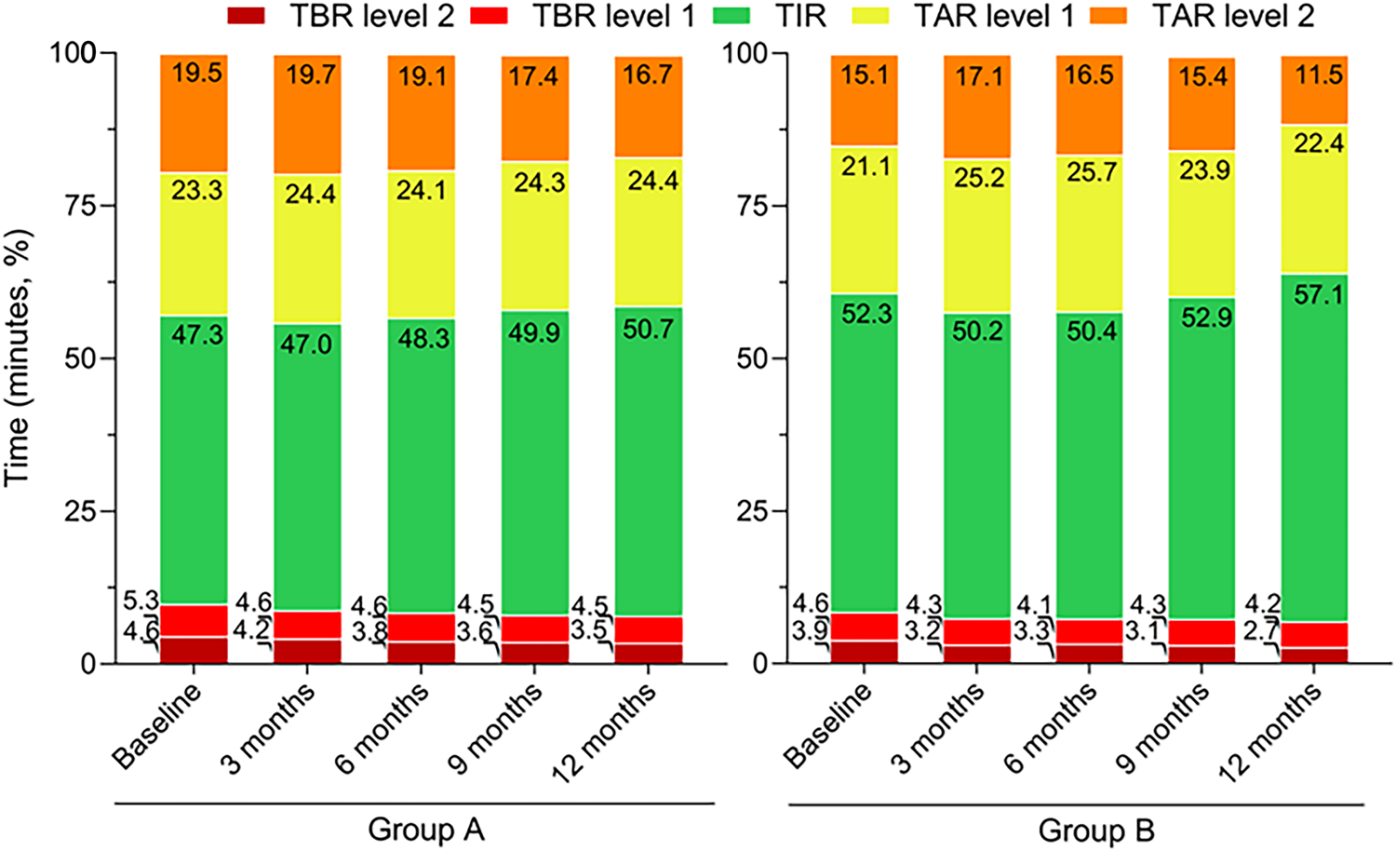
Time (minutes, %) in different glucose ranges: < 54 mg/dL (TBR level 2, very low, dark red), 54–69 mg/dL (TBR level 1, low, red), 70–180 mg/dL (TIR, target, green), 181–250 mg/dL (TAR level 1, high, yellow), e > 250 mg/dL (TAR level 2, high, orange) in the 2 groups (Group A-naive user for FGM device,left panel; Group B-former user for FGM device, right panel). TBR:time below range, TIR: time-in-range, TAR: time above range

No differences were observed between the two groups in the time spent in the different CGM metrics (Fig. 3). In group A, TBR level2 significantly decreased (*p* = 0.01) and TIR significantly increased in group A (*p* < 0.001) and group B *p* = 0.039 during the follow-up. TAR level 1, significantly increased in group A (*p* = 0.01), while TAR level 2 decreased in group A *p* = 0.048 and B *p* = 0.018 during 12-month.

In both groups the use of the isCGM was associated with a decrease in glucose variability (%CV) (*p* = 0.009) with a significant group*time interaction (p = 0.04). Group B showed a significantly lower %CV compared to group A at baseline, 3 and 12 months (supplementary Fig. 1a). As expected, in the whole population, subjects facing hypoglycemic episodes showed a higher glucose variability compared to those not experiencing hypoglycemia (*p* < 0.01). In the whole population, the percentage of time spent in TBR 1 and TBR 2 levels was highly correlated with CV% at all timepoints (*p* < 0.001) (data not shown).

HbA1c was significantly higher in group A compared to group B at baseline (*p* = 0.02) and showed a progressive significant time-dependent decrease in both groups (GLM, *p* < 0.001). In group A there was a significant decrease in HbA1c levels (*p* < 0.01) at all time points (supplementary Fig. 1b). The alignment between plasma HbA1c and estimated HbA1c -glucose management indicator- values was optimal (ρ > 0.80, *p* < 0.001) at all time-points (supplementary Fig. 2).

Significant predictors of HbA1c improvement were higher baseline HbA1c values (β = − 0.422, *p* < 0.01), a higher mean daily scan (*β* = − 0.393, *p* = 0.003) and younger age (β = − 0.08, *p* = 0.05) (data not shown). At baseline, in both study groups, individuals in CHO counting showed a lower baseline plasma HbA1C (*p* < 0.001). CHO counting was not associated to any changes in glucose metrics throughout the study.

### Diabetes Quality of life (secondary endpoint)

**Fig. 4:**
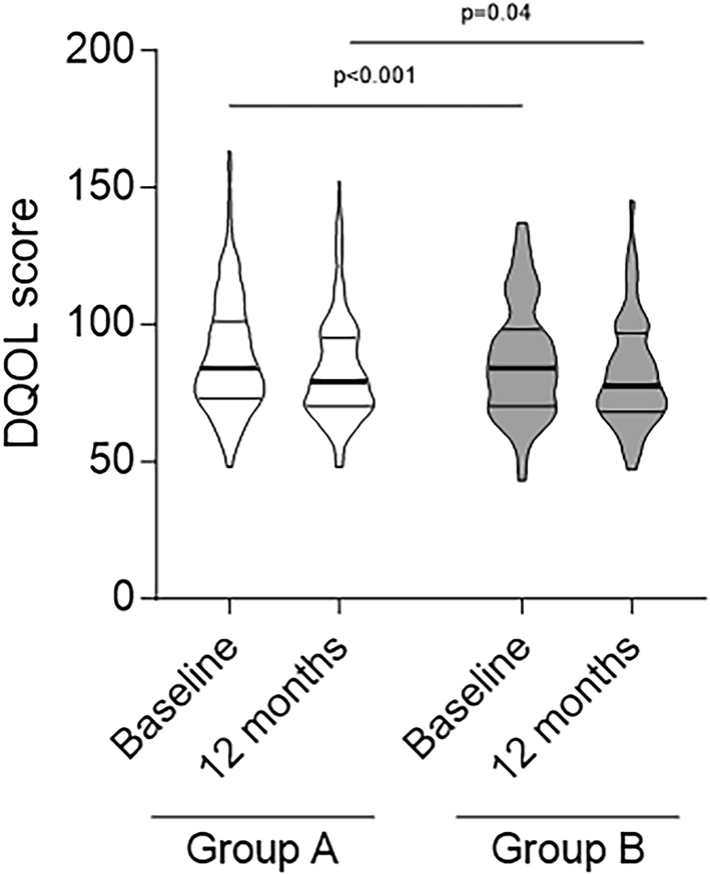
Diabetes quality of life (DQOL) score in the 2 groups (Group A-naive user for FGM device, white boxes; Group B-former user for FGM device, gray boxes) at baseline and 12 months. Truncated violin plots extend from the minimum and maximum values and median and quartiles are shown

DQOL satisfaction score decreased in both groups at 12 months (*p* < 0.001 and *p* = 0.04, respectively) without intergroup differences (*p* = 0.84) pointing to a significant improvement of the perceived well-being associated with the use of the isCGM system (Fig. 4).

### isCGM adherence and acceptability data

Daily mean number of sensor scans was 8 ± 4 in group A and 9.5 ± 4 in group B, without differences during follow-up. Device use expressed as percentage of data collected between visits was 84 ± 20% in both groups without differences throughout the study. The mean number of days/year CGM worn—338 ± 31 in group A (94%) and 343 ± 26 in group B (95%)- did not differ between groups. 130 subjects (39.6%) in group A and 35 (48.6%) in group B, experienced at least one sensor detachment and 24 subjects in group A (7.3%) and 7 in group B (9.7%) experienced patch allergic reactions without differences among groups (*p* = 0.92 and *p* = 0.87, respectively).

In the whole population there was a positive and significant correlation between the mean number of daily scans and TIR at all time-points (*p* = 0.014, *p* < 0.001, *p* < 0.001, *p* < 0.001 at 3, 6, 9 e 12 months, respectively) (data not shown).

In addition, the mean number of sensor scans was negatively associated with TBR level 2, TBR level 1 and TAR level 2 at all time points, while the association was significant for time spent with glucose > 180 mg/dL only at 3 months (data not shown).

## Discussion

This study showed the effectiveness of the isCGM in reducing SHE and in ameliorating glucose metrics and quality of life in a cohort of individuals with T1D in MDI therapy at high risk of acute diabetes complications, at least in the group of patients naïve to the device use.

Subjects with T1D recruited in this study are highly representative of the Italian T1D population as described in a large survey [19] as well as in other Italian cohorts with T1D [20].

The main study finding is the demonstration that the use of an isCGM in real-world conditions in a regional public health service scenario, reduced the incidence of SHE within 3 months and TBR < 3.0 mmol/L in a T1D naïve to the device. The lack of a significance reduction in already users may reconcile with the temporal shift which makes the effect no longer detectable.

Our results confirm those of RCTs conducted in well-controlled T1D [7] whereas in real-life studies these results are less neat. The use of isCGM in T1D has been associated either to a marked reduction in SHE and DKA [21], or to an increase in mild hypoglycaemic events [22], to no increase in SHE [23], to a reduction in SHE or comas and TBR at the expenses of a reduction in TIR and more time spent in hyperglycemia [24]. In a recent crossover trial, the TBR reduction with the use of isCGM device with structured education was associated with the increase in TAR level 2 compared to the SMBG group [25].

These results substantially differ from those in our study in which the reduction in hypoglycemic events was associated to a significant improvement in TIR and, importantly and consistently, in glucose variability, likely due to the high degree of education delivered in all patients.

The reduction in hypoglycemic events -obtained in the contest of a Regionwide reimbursement- is of extreme clinical relevance in relation to the robust association between hypoglycemic events and increased morbility and mortality in T1D [5, 26–28]. Of note, in our study, glucose variability- which was reduced in both groups with the isCGM use-, was directly associated with the occurrence of hypoglycemic events in line with previous studies [29, 30]. As expected, in line with the literature, the lack of a CHO counting diet regimen and a higher glucose variability -including a higher baseline plasma HbA1C levels- were associated to an increase in cumulative incidence of SHE [31]. Center effect may be due to regional intra-hospital differences in terms of diabetes management and team composition which is indicative of a real-world observation.

Our data confirm the effect of the isCGM in reducing CV% as showed in the IMPACT randomised study [9], widening the clinical evidence of the effectiveness of the system in ameliorating glucose metrics and the hypoglycemic risk. This was also confirmed by the early reduction in the plasma HbA1C in naïve patients, strengthening isCGM clinical importance in reducing the development and progression of microvascular and macrovascular complications [2, 3]. A meta-analysis of RWE confirmed that starting the FSL-1 system led to a significant and sustained reduction in HbA1c in T1D as a replacement for SMBG [32].

Patient adherence to the device resulted high (~ 95%) as well as the number of daily scans. A higher adherence was associated with higher TIR during follow-up and inversely correlated with time above range and TBR level 2 at all timepoints in accordance with previous reports [29, 33, 34]*.*

Importantly, in our T1D population the use of the isCGM significantly improved quality of life which is often a neglected aspect, but it is a key and compelling need in the care of T1D. Conversely, in the IMPACT study no differences emerged in DQOL score between intervention and control group [9].

We acknowledge some study limitations. As a real-life study several patients and data were lost in follow-up and the two groups were numerically unbalanced. However, the prospective nature and the relatively high number of patients representative of the Italian T1D population in a multicentric setting ensure the generalizability of the results.

Information on insulin doses or basal/bolus ratio which may influence SHE risk or glucose meters were not collected and included in the analysis.

Although, switching from isCGM to real time CGM significantly improved several glucose endpoints and reduce the risk of hypoglycemia [35, 36] at 6-month at least in T1D well experienced with the use of isCGM, further studies are warranted to confirm these results in naïve individuals and in a real-world setting and in a sustainability perspective [37].

In conclusions, our real-world study confirms and complements RCT data showing the effectiveness of the isCGM in reducing hypoglycemic risk without glucose deterioration. These results are clinically relevant considering the strong association between hypoglycemia and risk of mortality and that between glucose metrics and variability with micro and macro complications.

## Supplementary Information

Supplementary file 3 (TIF 100 kb)

Supplementary file 3 (TIF 179 kb)
