# Supplementary figures and images for: Effectiveness of the flash glucose monitoring system in preventing severe hypoglycemic episodes and in improving glucose metrics and quality of life in subjects with type 1 diabetes at high risk of acute diabetes complications
Source: Acta Diabetol. 2024 Jun 4;61(9):1177–84. doi: 10.1007/s00592-024-02298-x (PMC11379770; doi:10.1007/s00592-024-02298-x)

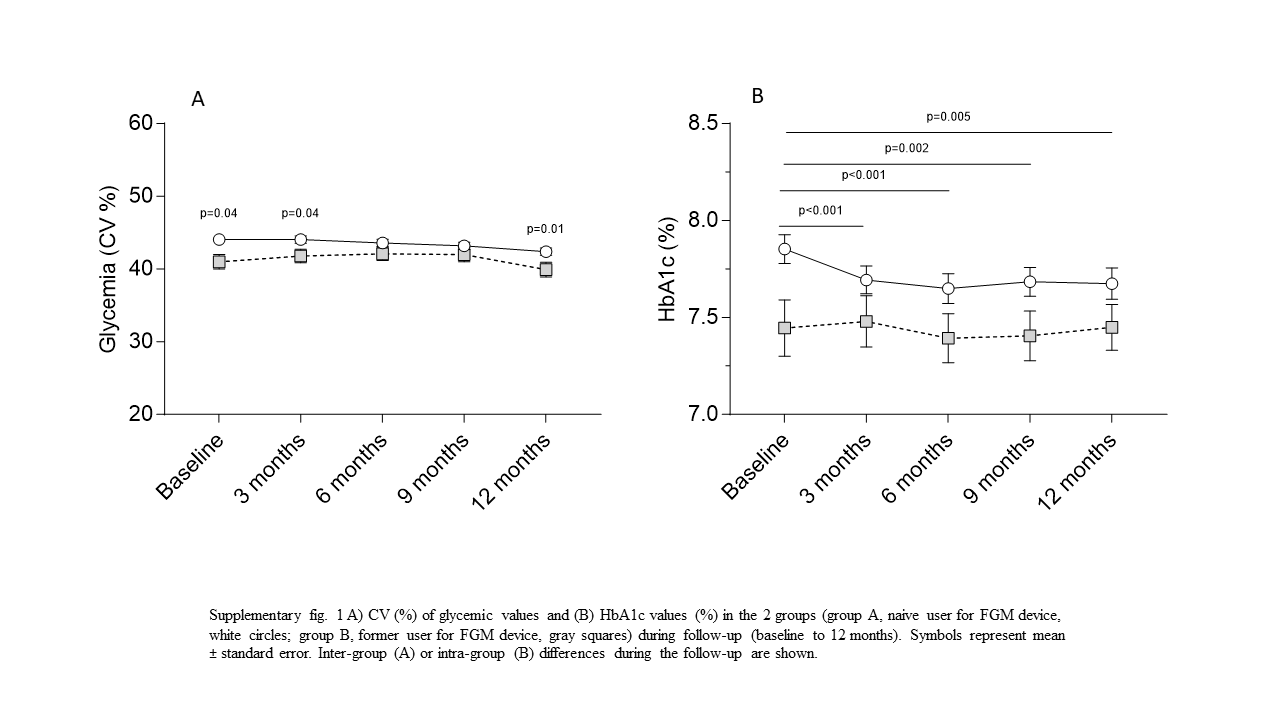

Supplement: Supplementary file 1 — Supplementary file 3 (TIF 100 kb) [file 592_2024_2298_MOESM1_ESM.tif]

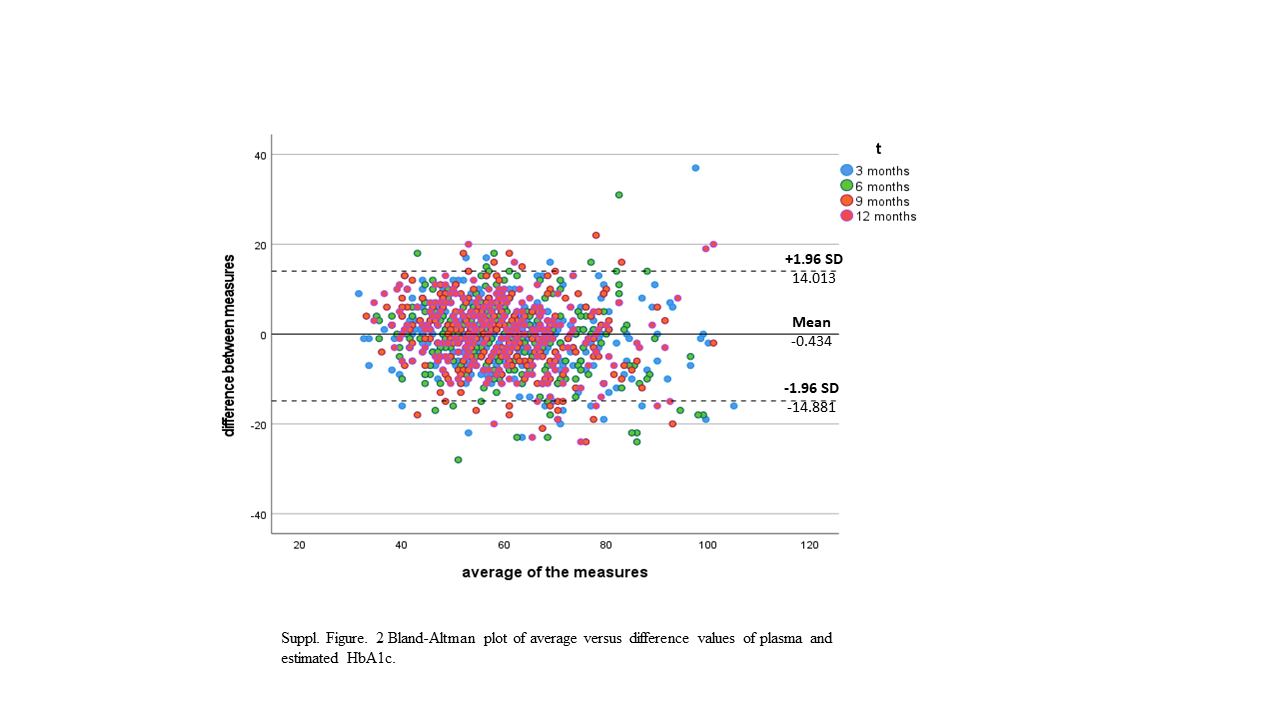

Supplement: Supplementary file 2 — Supplementary file 3 (TIF 179 kb) [file 592_2024_2298_MOESM2_ESM.tif]
